# Extracting

**Published:** 1881-10

**Authors:** William D. Kempton


### EXTRACTING.

### A PAPER READ BEFORE THE CINCINNATI DENTAL SOCIETY, SEPTEMBER 13, 1881.

BY WILLIAM D. KEMPTON, M.D., D.D.S.

*Mr. President and Gentlemen*:—At the present time, when so much stress is laid on the preservation of the natural teeth, and so much controversy as to the method of doing it is indulged in, the fact that teeth are sometimes extracted seems either to have been forgotten or regarded as beneath the notice of most of the contributors to our journals, but still the fact remains that teeth are extracted, and the bungling way in which it is often done has caused a great many timid people to resort to "gas."

The conditions where extracting is indicated are:

—Exodontosis,

—Unhealthy roots,

—To gain space in the correction of irregularity,

—Severe and continued pain with no prospect of speedy relief,

—Extensive caries of the first permanent molar if the second has not yet erupted,

—Teeth that have become loose from the loss of antagonists, or from the absorption of the process due to the presence of tartar or other irritants.

—Carious deciduous teeth, except canines, where the parents are not willing to have them filled, as the alveolar abscesses that are apt to occur, and the subsequent suppuration will undoubtedly disturb the nutrition of the permanent teeth beneath.

—Inflammation of the pulp or periodontium, in cases where the patient through indifference or ortherwise, fails to co-operate with the dentist in the treatment, or in cases occuring in the advanced stages of Phthisis Pulmonalis, Cancer, or any incurable wasting disease.

Whilst in any of these conditions, a resort to the forceps is advisable; yet there are certain circumstances in which a modification of this proceedure is necessary.

In cases of exhaustion or of pregnancy where several teeth are affected, it is better if little or no pain is present, to postpone the operation, but if the pain is at all severe, and especially if complicated by alveolar abscess, it is better to extract at once, as there is less to be feared from the shock of the operation than from the continued pain and suppuration.

The next thing after having decided to extract, is to prepare the patient for the operation. If a child, place a diagonally folded napkin close up under the chin, and push the ends down between the chair and the patients back to prevent the clothing being soiled by blood or bloody water. If an adult and several teeth are to be extracted, it is best, especially if the patient is a nervous one, to administer Spts. Frumenti zij.

In this connection, it may be well to notice the foolish and reprehensible practice of extracting from twelve to fourteen, and even more teeth at one sitting. Seven at the farthest are enough, for it is sometimes months before there is complete recovery from the shock, and derangement of the nervous system following the removal of too many.

It is to be supposed, of course, that the patient occupies the dental chair during the operation. For this purpose the pedal lever chairs cannot be excelled, as the patient can be readily placed in such a position as to be entirely under the control of the dentist, and enable him to exert his strength to the best possible advantage, but as we are sometimes obliged to operate at a distance from the office, some substitute must be improvised. The patient may be seated on an ordinary straight-backed chair, whilst the operator stands behind the right side of it, with his left foot on a stool, thus making a head rest of his thigh.

With children it is often difficult to get and keep the mouth open. To accomplish this, procure a piece of brass tubing of a size to admit the middle finger and two inches long; with this shield, the finger can be introduced between the jaws, and the mouth kept open with the greatest of ease.

When extracting teeth from the upper jaw, the chair should be reclined so that the long axis of the centrals will be horizontal and elevated to a height to suit the operator.

In operating on a central, the head should be held firmly against the head-rest by placing the palm of the left hand on the forehead, allowing the points of the middle and ring fingers to extend over the cutting edges of the lateral, cuspid, and bicuspid, whilst the lip is held up by the index finger. If the neck of the tooth is not softened, forceps No. 13, S. S. White, should be applied and forced up under the gum to the process, thus separating the gum from the tooth, and at the same time getting a good hold of the neck, firm pressure should be made first backward and then forward, increasing the force with each motion till the tooth is loosened. Then, and not till then, should traction be made in connection with the antero-posterior motion.

The operator should be cool and collected and never in a hurry, for it is of more importance to get a tooth out with one effort than to get it out quickly, and if the patient should grasp the dentist's hands, all motion should cease and the patient calmly but firmly told to remove his hands, and no farther attempt made till he obeys, for the operator cannot know how much force he is exerting, and if he continue, might break the tooth. If caries has extended into the neck, and there is any doubt of its ability to withstand the pressure necessary to loosen it, vertical incisions should be made through the gum over the median line of the tooth anterior and posterior. The object of these incisions is the division of the thickened ring of gum which surrounds the neck and prevents the forceps from passing far enough up. Alveolar forceps Nos. 58 or 32, S. S. W., should be used, and forced up from $\frac{1}{16}$ to $\frac{1}{8}$ of an inch beyond the free margin of the process, care being taken that the inner or posterior beak is kept in contact with the process and not forced up too far, as there is danger of injuring the vessels and nerves that find exit through the anterior palatine foramen.

Sufficient pressure should be made to cut through the bone, then motion as described above, but more carefully, as too violent efforts might crush a tooth that could otherwise be coaxed out.

Laterals require about the same treatment as centrals. Except where they are small, crowded or irregularly situated, in which case No. 1, S. S. W., are better suited to the purpose.

For the deciduous incisors and canines, the forceps just referred to are indicated. If, however, from the extent of caries they cannot be used, a pair with an Ambler Tees' beak should be applied and forced up as far as possible, when slight rotation combined with a gentle antero-posteror motion, will often cause the tooth to slip up into the forceps. If the apices of the roots protrude through the gums, an incision should be made through the gum from the orifice to the free margin, and a spoon-shaped scaler inserted under the apex and the root lifted out.

The permanent cuspids require much the same treatment as the centrals, only a little more so. If the adjoining lateral and bicuspid have been extracted at the same sitting, the tooth may be easily removed by inserting the beaks of No. 32 into their respective sockets.

For bicuspids, the head must be turned more to one side, and the cheek held away by the middle finger, and the lip held up by the index finger, if the tooth is on the left side, and vice versa for those on the right, No. 40, S. S. W., should be used, pressure being made first inward, then outward. Occasionally after these teeth are loosened, it requires considerable traction to remove them. If from the extent of caries, the use of No. 40 might result in fracture, the gum should be cut as directed above and No. 58 or 32 applied and lateral motion made.

For molars, the head should be turned a little to the left for teeth on the right side, and a good deal to the right for those on the left side. No. 19, S. S. W., right and left are the best for these teeth. In adjusting them, the operator should be careful not to set them too far back, so that the point on the outer beak will ride on the convex surface of the posterior buccal root, instead of entering the bifurcation, for it might slip back into the space between the teeth, and "two birds be killed with one stone," this precaution is equally valuable in extracting lower molars.

After having forced the forceps up as far as possible, very firm pressure should be made inward till the tooth starts, then outward, and these two motions kept up in connection with traction till the tooth leaves its socket. If the buccal wall is much softened, the outer beak should be forced well up and outward pressure be made first. If caries has extended as far as the process, the gum should be cut and the outer beak forced up on the process about $\frac{1}{16}$ of an inch and manipulated as before. This practice is advisable in similar cases occuring in any teeth.

When the crown is gone, and the roots still connected, though softened, the gum should be cut over each root and No. 32 applied over the lingual and anterior buccal roots, forced up on the process, and inward pressure made till the buccal root starts. If the posterior one does not move also, the outer beak should be released and placed over it, and the same motion made. By this means we may often bring out the three roots at once, we are sure of one at least, and as the rest are loose they can be easily removed. Where the roots are separate and have been so for some time, No. 7 S. S. W. for the right and No. 2 S. S. W. for the left are indicated. Too much pressure must not be made, as these roots are easily crushed. If the roots, although separate, are in contact, or if they are above the surface of the gum to any extent, No. 76 S. S. W., with an Ambler Tee's beak can be often successfully used, thus avoiding any incisions in the gum. It must be borne in mind that as the inner surface of the beaks are smooth, traction is entirely out of the question, and rotation combined with lateral motion must be depended on.

Owing to the small size of the third molar, its location and generally conically shaped root, No. 32 are better adapted to it than No. 19.

The deciduous molars require about the same treatment as the permanent ones, except that the use of alveolar forceps is not admissible.

For the lower teeth, the chair should be tilted back considerably, thus obviating the necessity of the operator leaning over too much and assuming an unsteady position. The patient's head will be found to be low enough unless the operator be rather short, in which case he will be obliged to stand on a broad stool of suitable height.

The jaw should be held firmly in place, to avoid the danger of luxation by pressing it back with the three inner fingers of the left hand, while the lip is held out of the way by the index finger.

For the incisors, No. 14 S. S. W. should be used, and backward pressure made first. If the teeth are crowded or irregular, or if loose roots are present, No. 2 will answer better. If an alveolar forceps is needed, No. 52 S. S. W. should be selected.

For the canines and bicuspids, No. 14 should be used for the left, and No. 37 Codman and Shurtleff, for the right, and inward pressure made first.

In extracting canines, where the second bicuspid has been absent for some time, the operator should proceed cautiously, as the process on the posterior surface of the first bicuspid having been partially absorbed, it has not a very firm support, and if the apex of the canines should curve very much backward, there is danger of lifting the bicuspid out with the canine.

If alveolar forceps are needed, No. 52 will answer for the left side and for the right canine, but for the right bicuspids, the operator should occupy a position as much in front of the patient as possible, and while supporting the jaw with the palm and thumb of the left hand, and holding the cheek away with the index finger, he should apply No. 32 and make lateral motion. If loose roots are present No. 2 are indicated.

For molars, No. 28 R., S. S. W. should be used for either side. After they have been placed on the tooth they should be forced down and forcibly closed, which proceedure will often loosen the tooth. Motion should be made first inward, then outward, and as soon as the tooth starts, traction in connection with these motions should be resorted to, but the traction force should not be too great lest the tooth leave its socket too suddenly, allow the forceps to strike the upper teeth and mayhap chip a corner off of an incisor.

If the caries has extended so far that these forceps cannot be used, No. 52 can be applied over the anterior root after the gum has been cut over both roots. In this way both roots may be brought out together, but if not, the remaining one will be somewhat loosened and can be easily secured.

When loose roots are present, No. 2 should be used, and when operating on the right side the same position should be assumed as when operating on bicuspid roots with No. 32. The deciduous teeth require about the same treatment as the corresponding teeth in the permanent set.

The above remarks apply to the majority of teeth, but we meet with some that have a peculiarly muddy appearance that are almost as brittle as glass, and it behooves the operator to be on the lookout for just such teeth, for they break when he is least expecting it, and put him to a good deal of trouble to get the roots out.

Where the roots are exposed and denuded of periosteum almost to their apices, they are generally fragile, and although seemingly very loose, are liable to fracture if roughly handled.

The operator should be careful when applying the forceps to avoid pinching the lip or catching the beard in the joint.

The gum should not be cut when it is possible to succeed without it. especially in children, as in their wrought up condition it may put them out of the notion of having the tooth extracted, but

> "If it were done, when 'tis done 'twere well
> It were done quickly——— "

The lance should be kept sharp by a frequent acquaintance with an oil stone.

It is well to have a supply of papers about four inches square on which to lay the teeth after extracting.

It is advisable to have the case containing the forceps so situated that on opening the drawer the patient cannot see the contents, and to avoid any needless display of instruments, as it has a bad effect on the patient in his excited condition.

It is a good idea, while the patient is occupied in rensing his mouth, to critically examine the tooth and remove any adherent portions of process, as most patients have a horror of having their "jaw" broken.

Finally, be sure to clean the instrument after each operation, and if it can be done before the patient leaves the office, so much the better, as it impresses him with the fact that you keep your instruments clean, and it furthermore prevents the possibility of innoculating innocent persons with the blood of some syphilitic patient previously operated on.

